# The effects of idebenone on mitochondrial bioenergetics

**DOI:** 10.1016/j.bbabio.2011.10.012

**Published:** 2012-02

**Authors:** Valentina Giorgio, Valeria Petronilli, Anna Ghelli, Valerio Carelli, Michela Rugolo, Giorgio Lenaz, Paolo Bernardi

**Affiliations:** a Consiglio Nazionale delle Ricerche Institute of Neurosciences and Department of Biomedical Sciences, University of Padova, Padova, Italy; b Department of Evolutionary and Experimental Biology, University of Bologna, Bologna, Italy; c Department of Neurological Sciences, University of Bologna, Bologna, Italy; d Department of Biochemistry “G. Moruzzi”, University of Bologna, Bologna, Italy

**Keywords:** CRC, calcium retention capacity, Cs, cyclosporin, DMEM, Dulbecco's modified Eagle's medium, Δψ_m_, mitochondrial membrane potential difference, DTT, dithiothreitol, FCCP, carbonylcyanide-*p*-trifluoromethoxyphenyl hydrazone, LHON, Leber's hereditary optic neuropathy, MELAS, mitochondrial encephalomyopathy with lactic acidosis and stroke-like episodes, MOPS, 4-morpholinepropanesulfonic acid, NQO1, NAD(P)H:quinone oxidoreductase 1, OCR, oxygen consumption rate, PTP, permeability transition pore, ROS, reactive oxygen species, TMRM, tetramethylrhodamine methyl ester, Mitochondria, Idebenone, Permeability transition, Electron transfer, ATP synthesis

## Abstract

We have studied the effects of idebenone on mitochondrial function in cybrids derived from one normal donor (HQB17) and one patient harboring the G3460A/MT-ND1 mutation of Leber's Hereditary Optic Neuropathy (RJ206); and in XTC.UC1 cells bearing a premature stop codon at aminoacid 101 of MT-ND1 that hampers complex I assembly. Addition of idebenone to HQB17 cells caused mitochondrial depolarization and NADH depletion, which were inhibited by cyclosporin (Cs) A and decylubiquinone, suggesting an involvement of the permeability transition pore (PTP). On the other hand, addition of dithiothreitol together with idebenone did not cause PTP opening and allowed maintenance of the mitochondrial membrane potential even in the presence of rotenone. Addition of dithiothreitol plus idebenone, or of idebenol, to HQB17, RJ206 and XTC.UC1 cells sustained membrane potential in intact cells and ATP synthesis in permeabilized cells even in the presence of rotenone and malonate, and restored a good level of coupled respiration in complex I-deficient XTC.UC1 cells. These findings demonstrate that idebenol can feed electrons at complex III. If the quinone is maintained in the reduced state, a task that in some cell types appears to be performed by dicoumarol-sensitive NAD(P)H:quinone oxidoreductase 1 [Haefeli et al. (2011) PLoS One 6, e17963], electron transfer to complex III may allow reoxidation of NADH in complex I deficiencies.

## Highlights

► The mitochondrial effects of idebenone in living cells are poorly characterized. ► Idebenone can be toxic or beneficial depending on its oxidation-reduction state. ► Idebenone is toxic through opening of the permeability transition pore. ► Idebenol stimulates respiration and ATP synthesis in cells lacking complex I. ► Short-chain quinones can be developed for the treatment of complex I defects.

## 1 Introduction

Coenzyme Q is a lipophilic molecule mostly, but not exclusively localized in the inner mitochondrial membrane [1,2]. It is composed of a redox active benzoquinone ring conjugated to an isoprenoid side chain whose length may differ among species. In man ubiquinone contains predominantly 10 isoprenyl units and is designated CoQ10. CoQ10 shuttles electrons from complex I and II and other flavoprotein dehydrogenases to complex III of the mitochondrial respiratory chain; it also functions as a lipid-soluble antioxidant, scavenges reactive oxygen species (ROS), and is involved in multiple aspects of cellular metabolism [1,2].

Primary CoQ10 deficiencies are associated with pathogenic mutations affecting genes involved in the biosynthesis of CoQ10, and cause clinically heterogeneous diseases [2]. Respiratory chain and ATP synthesis defects, ROS production, and apoptosis contribute to disease pathogenesis, and most patients affected by primary CoQ10 deficiencies respond to CoQ10 supplementation [1,3,4]. Because of the prominent role attributed to ROS in the pathogenesis of mitochondrial diseases, including Friedreich's ataxia; and because of the antioxidant effect of CoQ10 [5], its possible use in these diseases has been considered [1,6,7]. Given the fact that CoQ10 is very insoluble, several short-chain hydrosoluble quinones have been tested, in particular 2,3-dimethoxy-5-methyl-6-(10-hydroxydecyl)-1,4-benzoquinone (idebenone). Idebenone did not prove useful in CoQ10 deficiencies [4]; yet, due to its redox properties and the evidence that it can mediate electron transfer to respiratory chain complex III in isolated mitochondria [1,8–12], idebenone has been used in several diseases associated to respiratory chain dysfunction like Leber's hereditary optic neuropathy (LHON) [13–17], mitochondrial encephalomyopathy with lactic acidosis and stroke-like episodes (MELAS) [18–20], Leigh syndrome [21], Friedreich's ataxia [6,7,22,23], as well as Huntington's [24] and Alzheimer disease [25–28]. More recently, a double blind, placebo-controlled, randomized trial with idebenone in LHON patients (RODHOS study) has shown some efficacy [29], as also reported in the retrospective analysis of a large case series of LHON patients treated with idebenone during the acute phase of the disease [30].

Protective effects of idebenone against cell death induced by glutathione depletion have been reported in CEM leukemia cells [31] and skin fibroblasts from Friedreich's ataxia patients [32], as well as in staurosporine-induced apoptosis of cultural retinal neurons [33]; yet higher doses of idebenone were cytotoxic to skin fibroblasts derived from both Friedreich's ataxia patients and healthy donors [34]. The recent demonstration that idebenone undergoes reduction by cytosolic dicoumarol-sensitive NAD(P)H:quinone oxidoreductase 1 (NQO1) [35] provides a rationale for its use in complex I-deficient cells, yet potentially toxic effects of idebenone may depend on inhibition of complex I itself [8–12] and on opening of the mitochondrial permeability transition pore (PTP) [36], an inner membrane high-conductance channel involved in cell death [37].

The effects of idebenone have been studied surprisingly little in cells, in spite of their obvious importance for the potential therapeutic use of this drug. Here, we present a characterization of the mitochondrial effects of idebenone in human osteosarcoma-derived cybrids bearing wild-type mtDNA (HQB17) or the missense G3460A/MT-ND1 mutation of LHON (RJ206) [38,39]; and in XTC.UC1 cells bearing a truncating mutation in the same gene that hampers complex I assembly [40,41].

## 2 Materials and methods

### 2.1 Materials

Oligomycin, rotenone, antimycin A, malonate, malate, pyruvate, carbonylcyanide-*p*-trifluoromethoxyphenyl hydrazone (FCCP), decylubiquinone, dithiothreitol (DTT), ATP monitoring kit, P^1^,P^5^ - di(adenosine-5′) pentaphosphate pentasodium and protease inhibitors were from Sigma (Milan, Italy). Idebenone was from Apin Chemicals LTD (Oxon, UK). CsA was purchased from Calbiochem, whereas CsH was a generous gift of Dr. Urs Ruegg, Geneva. Calcium Green 5N was from Invitrogen (Milan, Italy). Tetramethylrhodamine methyl ester (TMRM) was purchased from Molecular Probes (Eugene, OR).

### 2.2 Isolation of mitochondria and swelling measurements

Mouse liver mitochondria were isolated in a buffer containing 250 mM sucrose, 10 mM Tris, 0.1 mM EGTA, pH 7.4, as described previously [42]. Swelling was monitored as the decrease of 90° light scattering of the mitochondrial suspension at 545 nm with a Perkin-Elmer 650–40 fluorescence spectrofluorimeter equipped with magnetic stirring and thermostatic control.

### 2.3 Measurements of calcium retention capacity

The mitochondrial Ca^2 +^ retention capacity (CRC) was determined by measuring external Ca^2 +^ following addition of 10 μM Ca^2 +^ pulses each minute to medium containing 0.5 mg/ml of mitochondria. External Ca^2 +^ concentration was measured fluorimetrically in the presence of 1 μM Calcium Green 5 N, a membrane impermeant Ca^2 +^ probe increasing its fluorescence upon Ca^2 +^ binding (excitation and emission wavelengths 503 and 525 nm, respectively). The CRC is the amount of Ca^2 +^ taken up by mitochondria before the precipitous release that follows occurrence of the permeability transition. The CRC values observed at a given concentration of idebenone were normalized to the CRC obtained in the absence of the compound (CRC_0_).

### 2.4 Cell culture and growth conditions

HQB17 and RJ206 cells cybrids were generated by fusion of enucleated fibroblasts derived from one control (HQB17) and one LHON patient (RJ206, a kind gift of Anthony H. Schapira) into osteosarcoma 143B.Tk^−^ cells deprived of their own mtDNA [43]. Complete mtDNA sequencing revealed that HQB17 belongs to haplogroup J1b (Antonio Torroni, personal communication). RJ206 cells harbor the G3460A/MT-ND1 LHON mutation causing the A52T amino acid substitution in MT-ND1 [38,39], which was reported to sharply reduce mitochondrial ATP synthesis driven by complex I substrates [44]. XTC.UC1 cells, derived from a human thyroid carcinoma, bear a C insertion at bp3571 in MT-ND1, generating a premature stop codon at amino acid 101 of ND1 subunit that prevents complex I assembly [40,41]. Cells were grown in Dulbecco's modified Eagle's medium (DMEM) containing 10% fetal bovine serum, 2 mM L-glutamine, 100 units/ml penicillin, and 100 g/ml streptomycin in a humidified incubator at 37 °C with 5% CO_2_.

### 2.5 Mitochondrial membrane potential

**Fig. 1:**
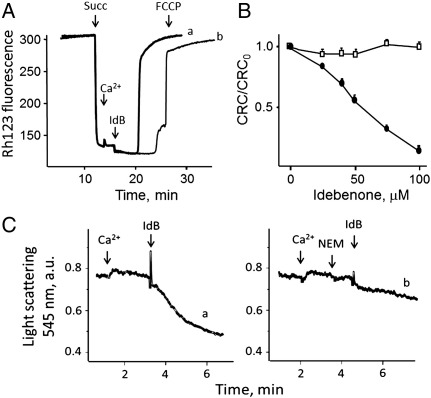
Effects of idebenone on mitochondrial membrane potential, Ca^2 +^ retention capacity and volume. *A*, the incubation medium contained 130 mM KCl, 10 mM MOPS-Tris, 1 mM Pi-Tris, 10 μM EGTA, 0.15 μM Rhodamine 123, pH 7.4 and 0.8 μM CsA (*trace b* only); where indicated, 5 mM succinate (Succ), 35 μM Ca^2 +^, 50 μM idebenone (IdB) and 0.5 μM FCCP were added. *B*, the mitochondrial CRC was determined following the addition of 10 μM Ca^2 +^ pulses, and values were normalized to the CRC obtained in the absence of idebenone (CRC_0_). Mitochondria were treated with the indicated concentrations of idebenone in the absence (*closed symbols*) or presence (*open symbols*) of 1 mM DTT. *C*, the incubation medium contained 0.25 M sucrose, 1 mM Pi-Tris, 10 mM MOPS-Tris, 20 μM EGTA-Tris, 5 mM glutamate-Tris, 2.5 mM malate-Tris. Where indicated 50 μM Ca^2 +^, 20 μM N-ethylmaleimide (NEM) and 50 μM idebenone (IdB) were added.

Mitochondrial membrane potential (Δψ_m_) in isolated organelles was measured using a Perkin-Elmer LS50B spectrofluorometer and evaluated based on the fluorescence quenching of Rhodamine 123. Mitochondria (0.5 mg/ml) were added to 2 ml of 130 mM KCl, 10 mM MOPS-Tris, 1 mM Pi-Tris, 10 μM EGTA, 0.15 μM Rhodamine 123, pH 7.4. The fluorescence of Rhodamine 123 was monitored at excitation and emission wavelengths of 503 and 523 nm, respectively, with the slit width set at 2.5 nm. After a short incubation to reach stabilization of the signal, further additions were as indicated in the legend to Fig. 1.

For measurements of Δψ_m_ in situ, cells were seeded onto 24 mm-diameter round glass coverslips and grown for 2 days in DMEM. Δψ_m_ was measured based on the accumulation of TMRM in the presence of 1.6 μM CsH, which inhibits the multidrug resistance pump but not the PTP [42,45]. Cells were incubated in bicarbonate- and phenol red-free Hank's balanced salt solution (Sigma) supplemented with 10 mM Hepes and 1.6 μM CsH and loaded with 20 nM TMRM for 30 min. At the end of each experiment, mitochondria were fully depolarized by the addition of 4 μM of the protonophore carbonylcyanide-*p*-trifluoromethoxyphenyl hydrazone (FCCP). Cellular fluorescence images were acquired with an Olympus IX71/IX51 inverted microscope equipped with a xenon light source (150 W) for epifluorescence illumination and with a digital camera. For detection of fluorescence 568 ± 25 nm bandpass excitation and 585 nm longpass emission filter settings were used. Images were collected with an exposure time of 100 ms (6% illumination intensity) using a 40X, 1.3 NA oil immersion objective (Olympus). Data were acquired and analyzed using Cell R software (Olympus). Clusters of several mitochondria (10–30) were identified as regions of interest, and fields not containing cells were taken as the background. Sequential digital images were acquired every minute, and the average fluorescence intensity of all relevant regions was recorded and stored for subsequent analysis.

### 2.6 Mitochondrial NAD(P)H

Cells were seeded as described above and incubated in bicarbonate- and phenol red-free Hank's balanced salt solution supplemented with 10 mM Hepes and 1.6 μM CsH for 30 min. Alamethicin (50 μM) was added at the end of each experiment to allow complete release of pyridine nucleotides from mitochondria. For detection of fluorescence 340 nm bandpass excitation and 465 nm longpass emission filter settings were used. Images were collected with an exposure time of 800 ms (100% illumination intensity) using a 40X, 1.3 NA oil immersion objective (Olympus).

### 2.7 Oxygen consumption rate

Oxygen consumption rate (OCR) in adherent cells was measured with an XF24 Extracellular Flux Analyzer (Seahorse Bioscience, Billerica MA, USA). HQB17 and RJ206 cybrids, and XTC.UC1 cells were seeded in XF24 cell culture microplates (Seahorse Bioscience) at 2 × 10^4^ cells/well in 200 μl of DMEM containing 4.5 g/l glucose (DMEM-high glucose) and incubated at 37 °C in 5% CO_2_ for 24 h. Assays were initiated by replacing the growth medium in each well with 670 μl of unbuffered DMEM-high glucose prewarmed at 37 °C. The cells were incubated at 37 °C for 30 min to allow temperature and pH equilibration. After an OCR baseline measurement, 70 μl of oligomycin, FCCP, rotenone and antimycin A were sequentially added to each well to reach final concentrations of 1 μg/ml for oligomycin, 0.2 μM (cybrids) or 0.1 μM (XTC.UC1 cells) for FCCP, and 1 μM for rotenone and antimycin A. Data are expressed as pmol of O_2_ per minute per 2 × 10^4^ cells. At the end of each experiment the medium was removed and cells were incubated with 0.1 μM calcein-AM in 200 μl/well of Hank's balanced salt solution supplemented with 10 mM Hepes and 1.6 μM CsH for 30 min, at 37 °C. Calcein-labeled adherent cells were observed with the Olympus IX71/IX51 inverted microscope (excitation and emission 495 and 515 nm, respectively, exposure time 100 ms, 6% illumination intensity) using a 10X, 1.3 NA oil immersion objective (Olympus).

### 2.8 Mitochondrial ATP synthesis

The rate of mitochondrial ATP synthesis was measured in digitonin-permeabilized cells incubated in 150 mM KCl, 25 mM Tris–HCl, 2 mM EDTA, 0.1% BSA, 10 mM K-Pi, 0.1 mM MgCl_2_, pH 7.4, in the presence of the inhibitor of adenylate kinase (0.1 mM P^1^,P^5^ -di(adenosine-5′) pentaphosphate) and of complex I substrates (5 mM malate and 5 mM pyruvate) by using the luciferin/luciferase assay [46], as detailed by the manufacturer's instructions. Addition of luciferin/luciferase (which according to Sigma is lyophilized with MgSO_4_) increases the Mg^2 +^ concentration to a level sufficient to allow activity of the enzyme, but still low enough to minimize the activity of oligomycin-insensitive ATPases. Thus, although the measured ATP synthesis rate was entirely of mitochondrial origin (as indicated by its full sensitivity to oligomycin) the actual figures may be underestimated because of the concomitant occurrence of ATP-hydrolyzing reactions. The reaction was started by addition of 0.1 mM ADP and chemiluminescence was determined as a function of time with a luminometer. The chemiluminescence signal was calibrated with an internal ATP standard after addition of 10 μM oligomycin. Data were normalized for citrate synthase activity [47]. Further additions are indicated in the figure legends.

### 2.9 Statistics

Unless otherwise stated in the figure legends, each experiment was repeated 3 times. Data are presented as average ± S.E. or, for clarity, as representative experiments (see figure legends for details).

## 3 Results

Following energization with succinate, mitochondria underwent a small and transient depolarization upon the addition of Ca^2 +^, which is the expected response to Ca^2 +^ accumulation (Fig. 1*A*). Subsequent addition of 50 μM idebenone triggered fast and complete mitochondrial depolarization (*trace a*) which was delayed and partially prevented by CsA (*trace b*), consistent with the PTP-inducing effects of idebenone [36]. Idebenone caused the expected decrease of the CRC [36], a sensitive measure of the propensity of the PTP to open; yet this effect was fully prevented by pretreatment of mitochondria with CsA (results not shown) or with DTT (Fig. 1*B*). The latter finding is novel and of some interest, because it suggests that idebenone may interact with a PTP regulatory site that is sensitive to oxidation-reduction events. This point was investigated further. Following accumulation of a permissive Ca^2 +^ load isolated mitochondria treated with idebenone underwent the expected PTP-mediated swelling response (Fig. 1*C*, *trace a*), while pore opening was prevented by treatment with a low concentration of N-ethylmaleimide (Fig. 1*C*, *trace b*), which we have shown to block PTP-regulating sulfhydryl groups that are also sensitive to reduction with DTT [48].

**Fig. 2:**
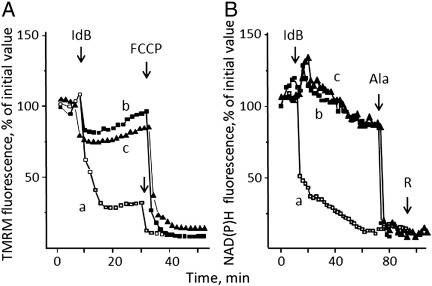
Effects of idebenone, CsA and decylubiquinone on mitochondrial TMRM accumulation and NAD(P)H levels. *A*, TMRM fluorescence and *B*, NAD(P)H fluorescence of HQB17 cells in the absence (*open symbols, traces a*) or presence of 1.6 μM CsA (*closed squares, traces b*) or 50 μM decylubiquinone (*closed triangles, traces c*). Where indicated 50 μM idebenone (IdB), 4 μM FCCP, 80 μM alamethicin (Ala) and 4 μM rotenone (R) were added. Data in both *panels* report one representative experiment of five (for idebenone and idebenone plus CsA) or three (idebenone plus decylubiquinone). The maximal S.E., which is omitted for clarity, was ± 10%.

We next tested the effects of idebenone on Δψ_m_ in intact cells based on accumulation of TMRM in HQB17 cybrids containing normal mtDNA. Addition of 50 μM idebenone was followed by a rapid drop of fluorescence of TMRM (Fig. 2*A, trace a*), which indicates fast mitochondrial depolarization. This fluorescence decrease was largely prevented by treatment with both CsA (Fig. 2*A, trace b*) and decylubiquinone (Fig. 2*A, trace c*), reagents that inhibit the PTP at different sites [49]. Since prolonged openings of the PTP are followed by membrane permeabilization to pyridine nucleotides [50], we also monitored mitochondrial NAD(P)H levels, which were rapidly decreased by the addition of idebenone (Fig. 2*B, trace a*) in a process that was prevented by CsA (Fig. 2*B, trace b)* or decylubiquinone (Fig. 2*B*, *trace c*) but still inducible with the pore-forming peptide alamethicin (Fig. 2*B*). Addition of rotenone after idebenone or alamethicin was not followed by NAD(P)^+^ reduction (Fig. 2*B*), indicating that PTP opening (or alamethicin addition) causes the release of mitochondrial pyridine nucleotides rather than just their oxidation, at variance from treatment with FCCP alone (results not shown). The PTP-inducing effects of idebenone could be due to increased production of ROS, which has been shown to occur upon treatement of submitochondrial particles with this compound [12,51].

**Fig. 3:**
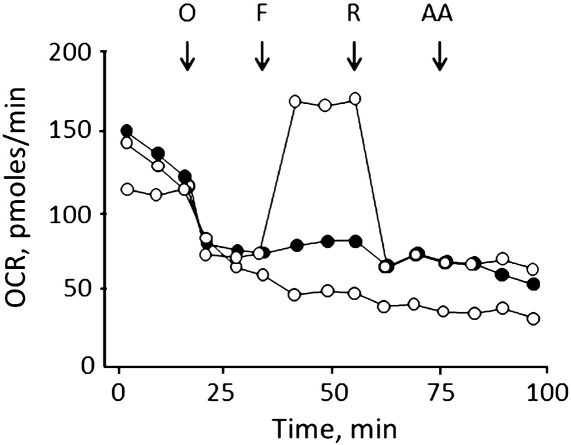
Effects of idebenone on respiration of HQB17 cells. HQB17 cells were incubated in Seahorse 24-well plates (20,000 cells/well) and respiration measured in the absence (*open circles*) or presence of 50 μM idebenone alone (*gray circles*) or with 1.6 μM CsA (*closed symbols*). Where indicated 1 μg/mL oligomycin (O), 0.2 μM FCCP (F), 1 μM rotenone (R), and 1 μM antimycin A (AA) were added. Data report one representative experiment of three, and the maximal S.E. was ± 11.3 pmol oxygen/min. OCR, oxygen consumption rate.

The bioenergetic consequences of treatment with idebenone were also assessed by measurements of oxygen consumption with the sensitive Seahorse technology. Respiration of HQB17 cells was substantially inhibited by oligomycin, indicating a good phosphorylation capacity; it was stimulated well above basal levels by uncoupler, indicating a large respiratory reserve; and it could be largely inhibited by rotenone with no further effects of antimycin A (Fig. 3, *open symbols*). Addition of 50 μM idebenone was followed by a decreased OCR, which could no longer be stimulated by FCCP (Fig. 3, *gray symbols*). The protective effects of CsA were negligible (Fig. 3, *closed symbols*), in spite of preservation of the pyridine nucleotide pool.

**Fig. 4:**
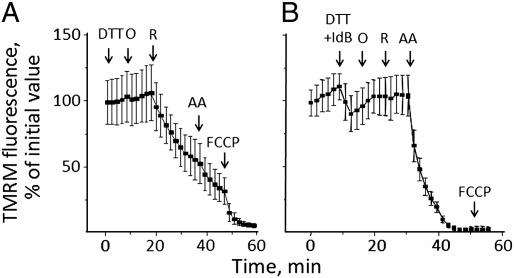
Effects of DTT and idebenone on mitochondrial TMRM fluorescence in HQB17 cells. HQB17 cells were loaded with 10 nM TMRM. *A*, additions were 2 mM DTT, 5 μM oligomycin (O), 4 μM rotenone (R), 1 μM antimycin A (AA) and 4 μM FCCP (F); *B*, additions were 2 mM DTT plus 50 μM idebenone (DTT + IdB), 5 μM oligomycin (O), 4 μM rotenone (R), 1 μM antimycin A (AA) and 4 μM FCCP. Data are from three or six independent experiments for *panels A* and *B*, respectively.

**Fig. 5:**
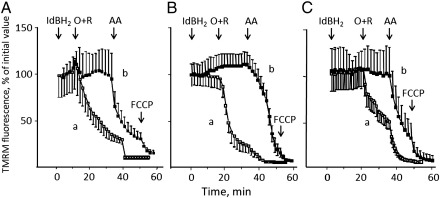
Effects of idebenol on mitochondrial TMRM fluorescence in HQB17, RJ206 and XTC.UC1 cells. HQB17 (*A*), RJ206 (*B*) and XTC.UC1 cells (*C*) were loaded with 10 nM TMRM in the absence (*open symbols*) or presence (*closed symbols*) of 1.6 μM CsA, and changes in fluorescence were monitored by fluorescence microscopy. Where indicated 50 μM idebenol (IdBH_2_), 5 μM oligomycin plus 4 μM rotenone (O + R), 1 μM antimycin A (AA) and 4 μM FCCP were added. Data are from three, eleven and ten independent experiments for *panels A*, *B*, and *C*, respectively.

We then tested whether the PTP-inducing effects of idebenone observed in isolated mitochondria could be prevented by reduction with DTT in cells as well. DTT alone was unable to sustain the membrane potential after addition of rotenone to oligomycin-treated HQB17 cells (Fig. 4*A*). Strikingly, however, the addition of DTT plus idebenone (i) prevented the depolarizing effects otherwise induced by the latter (Fig. 4*B*, compare with Fig. 2*A*); and (ii) allowed maintenance of the mitochondrial membrane potential after inhibition of complex I by rotenone, depolarization requiring the addition of antimycin A (Fig. 4*B*). These results are consistent with electron transfer to complex III. We assessed whether this effect could be also observed after treatment of cells with idebenol obtained by reduction of idebenone with sodium dithionite [52]. Treatment of HQB17 (Fig. 5*A*), RJ206 (Fig. 5*B*) or XTC.UC1 cells (Fig. 5*C*) with rotenone plus oligomycin caused a rapid depolarization (*open symbols, trace a* in all *panels*) suggesting that oxidation of idebenol (i.e. generation of idebenone) causes sensitization of the PTP. Indeed, in the presence of CsA the Δψ_m_ was preserved and depolarization was only observed after the addition of antimycin A (*closed symbols, trace b* in all *panels*), indicating electron feeding at complex III.

**Fig. 6:**
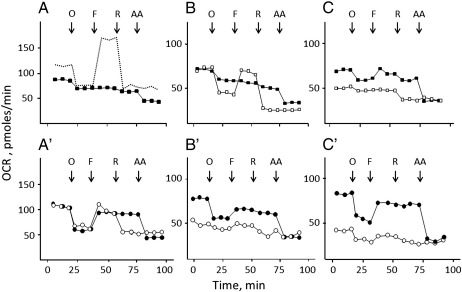
Idebenol and DTT-reduced idebenone promote basal respiration in XTC.UC1, but not in HQB17 and RJ206 cells. Cellular OCR of HQB17 (*A, A′*), RJ206 (*B,B′*) and XTC.UC1 cells (*C,C′*) was measured in 24-well Seahorse plates (20,000 cells/well). Cells were incubated in the absence of added quinone (*open squares*; the *dashed trace* in panel A is taken from Fig. 3). *A–C*, cells were supplemented with 50 μM idebenol (*closed squares*); *A′-C′*, cells were supplemented with 1 mM DTT (*open circles*) or 1 mM DTT plus 50 μM idebenone (*closed circles*). Note the different scale in *panels A* and *A′*. Data are representative of at least seven independent experiments, and the maximal variation (in pmol oxygen/min) was 12.5 (*panel A*), 3.1 (*panel B*), 7.5 (*panel C*), 9.9 (*panel A′*), 4.5 (*panel B′*), 4.3 (*panel C′*).

The next questions we addressed were whether idebenol can improve respiratory performance in cells where activity of complex I is compromised, and whether inhibition of complex I occurs in cells with a normal respiratory function. To answer these questions we studied respiration of HQB17 (Fig. 6*A,A′*), RJ206 (Fig. 6*B,B′*) and XTC.UC1 cells (Fig. 6*C,C′*).

In HQB17 cells the addition of idebenol decreased basal respiration, which showed virtually no response to oligomycin, FCCP and rotenone (Fig. 6*A*; compare with *dashed trace* for the same experiment carried out in the absence of idebenol, data taken from Fig. 3). This behavior presumably reflects PTP opening by idebenone which forms during idebenol oxidation (Fig. 2, see also below). Indeed, addition of DTT plus idebenone had a marginal effect on basal respiration, which maintained sensitivity to oligomycin (Fig. 6*A′*). OCR could not be stimulated further by FCCP, but it became insensitive to rotenone while it could be inhibited by antimycin A, consistent with direct electron transfer to complex III (Fig. 6*A′*). DTT alone inhibited maximal respiration, suggesting that the inhibitory effect on complex I may be caused by DTT rather than idebenol. In the absence of idebenol DTT was unable to sustain respiration after addition of rotenone (Fig. 6*A′*).

RJ206 cells displayed a lower OCR than wild-type cells and a normal response to oligomycin, but could not be stimulated beyond the initial rate by FCCP, indicating no reserve respiratory capacity (Fig. 6*B*). Like in HQB17 cybrids, treatment with idebenol made respiration totally insensitive to oligomycin, FCCP and rotenone (Fig. 6*B*). Also in this case, DTT plus idebenone allowed coupled (i.e. oligomycin-sensitive) respiration to occur in spite of a marked toxicity of DTT alone (Fig. 6*B′*).

XTC.UC1 cells slightly improved their performance after the addition of idebenol, and displayed a very remarkable increase of the oligomycin-sensitive (i.e., phosphorylating) OCR with DTT plus idebenone in spite of the inhibitory effect of DTT alone (Fig. 6*C′*).

**Fig. 7:**
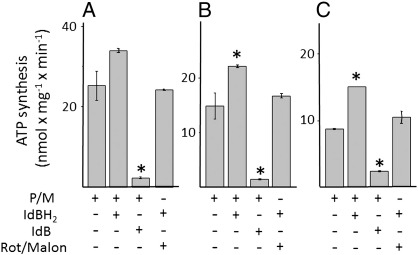
ATP synthesis in permeabilized HQB17, RJ206 and XTC.UC1 cells. HQB17 (*A*), RJ206 (*B*) and XTC.UC1 (*C*) cells were permeabilized with digitonin and rate of ATP synthesis was evaluated in the presence of 5 mM pyruvate and 5 mM malate (P/M), 50 μM idebenol (IdBH_2_), 50 μM idebenone (IdB), 5 μM rotenone and 5 mM malonate (Rot/Malon) as indicated. Asterisks denote values significantly different from P/M alone (P < 0.05).

We finally directly assessed the effects of idebenone and idebenol on ATP production with different substrates and inhibitors. It should be noted that in these experiments no Ca^2 +^ was added thus preventing PTP opening which is strictly Ca^2 +^-dependent. Remarkably, idebenol improved ATP synthesis in all cell types, and it allowed oxidative phosphorylation to proceed after inhibition of complex I with rotenone and of succinate dehydrogenase with malonate (Fig. 7). The rate of ATP synthesis could not be stimulated further by glycerol-3-phosphate (results not shown).

## 4 Discussion

In this study we have characterized the effects of idebenone and idebenol on mitochondrial bioenergetics. We have shown (i) that idebenone induces PTP opening in isolated mitochondria [36] through a redox-sensitive site that can be protected by N-ethylmaleimide or by reduction with DTT, an observation that proved instrumental in dissecting the complex effects of idebenone and idebenol in intact cells; (ii) that idebenone induces PTP opening also in intact HQB17 cells; under these circumstances idebenone does not mediate electron transfer between NAD(P)H and respiratory complex III; and (iii) that in the presence of DTT added idebenone (which presumably is fully reduced to idebenol) does not have PTP-inducing effects in cells and it allows rotenone-insensitive electron transfer to complex III of the respiratory chain, in a process that profoundly ameliorates the bioenergetic performance of XTC.UC1 cells lacking complex I. Interestingly, idebenol has mixed effects, i.e. it can sensitize the PTP and/or cause respiratory inhibition presumably because of the idebenone generated by its oxidation, as also shown by sensitization of the PTP to opening (which can be revealed by depolarization following the addition of oligomycin and rotenone). These results are novel because the effects of idebenone on mitochondrial bionergetics (specifically, the possible occurrence of PTP opening) had never been assessed in intact cells; and they are relevant to LHON, a maternally inherited disease due to primary mitochondrial DNA mutations affecting complex I [53].

Reduced ATP synthesis and increased oxidative stress are believed to sensitize retinal ganglion cells to apoptosis, which leads to blindness [54–57]. Recent evidence suggests that the PTP may be involved in the pathophysiology of this disease [41], and the possible occurrence of a permeability transition following treatment with idebenone is therefore of major concern, particularly in view of the fact that the preparation used in clinical trial is the oxidized form of the drug. Our finding that DTT plus idebenone is free of PTP-inducing effects and allows rotenone-insensitive electron transfer to complex III is therefore of interest to the development of treatments useful to bypass the defects of complex I.

Idebenone strongly activates glycerol phosphate oxidation in brown adipose tissue mitochondria, an effect that could be traced to release of the inhibition of glycerol phosphate dehydrogenase by endogenous free fatty acids [58]. We did not observe stimulation of ATP production by glycerol-3-phosphate in digitonin-permeabilized HQB17, RJ206 and XTC.UC1 cells treated with idebenone (results not shown), suggesting that idebenone is not reduced by glycerol phosphate dehydrogenase in these cells. The possibility that this occurs in other cell types must, however, be borne in mind.

A pathway for electron transfer from NADH to mitochondria has recently been identified with the demonstration that idebenone is a good substrate of cytosolic dicoumarol-sensitive NQO1 [35]; and that rescue of ATP levels by idebenone in different cell types correlated with expression of NQO1, which may provide an effective electron transfer from cytosolic NAD(P)H to idebenone and then complex III, thus reducing lactic acidosis in patients with complex I deficiency [35]. Remarkably, administration of 400 mg/kg/day of idebenone to mice for 4 weeks significantly slowed down the depletion of ATP induced by rotenone in isolated hepatocytes that had not been further treated with the drug [35]. In our cell lines rotenone-insensitive respiration in the presence of idebenol was not inhibited by dicoumarol (results not shown), suggesting that idebenone is not reduced by NQO1. This is consistent with PTP sensitization and inhibition of respiration in the course of idebenol oxidation, which we explain with generation of idebenone (Figs. 5 and 6).

Besides anecdotal reports on the effects of idebenone therapy in various conditions [13–17], the largest experience has been on patients with LHON both through a prospective controlled trial [29] and a retrospective re-evaluation of large cohorts of treated patients [30]. These studies revealed some efficacy although a subclass of patients is poor responders [29,30]. The pathogenic mechanism of the different LHON mutations, which has not been fully elucidated yet, may be relevant to the variable success of idebenone in compensating complex I dysfunction. Furthermore, it should be mentioned that genetic variation in NQO1 [59,60], as well as in mtDNA haplogroups potentially regulating respiratory chain coupling and antioxidant enzymes, are putative modifiers of the therapeutic response to idebenone in LHON.

The in vivo studies provided encouraging clinical evidence that idebenone is safe and well tolerated. Since idebenone is transformed into its metabolites QS-10, QS-6 and QS-4 within minutes of administration, and parent idebenone is not detectable in plasma after about 1 h [22,61], we suspect that the beneficial effects are not due to idebenone itself but to one of its longer-lasting metabolites. An attractive candidate appeared to be the carboxy derivative QS-10, which is a good electron acceptor from NQO1; yet QS-10 failed to restore ATP levels and to decrease lactate production in MELAS cells [35]. It will be important to test the mitochondrial effects of idebenone metabolites in order to identify the active species, a strategy that should provide effective and safer compounds devoid of the potentially toxic PTP-inducing properties of idebenone [36,62].
